# Prior knowledge auxiliary for few-shot pest detection in the wild

**DOI:** 10.3389/fpls.2022.1033544

**Published:** 2023-01-26

**Authors:** Xiaodong Wang, Jianming Du, Chengjun Xie, Shilian Wu, Xiao Ma, Kang Liu, Shifeng Dong, Tianjiao Chen

**Affiliations:** ^1^ Institute of Intelligent Machines, Hefei Institutes of Physical Science, Chinese Academy of Sciences, Hefei, China; ^2^ Science Island Branch, Graduate School of University of Science and Technology of China, Hefei, China; ^3^ Department of Automation, University of Science and Technology of China, Hefei, China; ^4^ School of Computer Science and Engineering, Nanjing University of Science and Technology, Nanjing, China; ^5^ Department of Computer Science, University of Sheffield, Sheffield, United Kingdom

**Keywords:** few-shot detection, hierarchical structure, pest recognition, prior knowledge, cross-relation

## Abstract

One of the main techniques in smart plant protection is pest detection using deep learning technology, which is convenient, cost-effective, and responsive. However, existing deep-learning-based methods can detect only over a dozen common types of bulk agricultural pests in structured environments. Also, such methods generally require large-scale well-labeled pest data sets for their base-class training and novel-class fine-tuning, and these significantly hinder the further promotion of deep convolutional neural network approaches in pest detection for economic crops, forestry, and emergent invasive pests. In this paper, a few-shot pest detection network is introduced to detect rarely collected pest species in natural scenarios. Firstly, a prior-knowledge auxiliary architecture for few-shot pest detection in the wild is presented. Secondly, a hierarchical few-shot pest detection data set has been built in the wild in China over the past few years. Thirdly, a pest ontology relation module is proposed to combine insect taxonomy and inter-image similarity information. Several experiments are presented according to a standard few-shot detection protocol, and the presented model achieves comparable performance to several representative few-shot detection algorithms in terms of both mean average precision (mAP) and mean average recall (mAR). The results show the promising effectiveness of the proposed few-shot detection architecture.

## 1 Introduction

Food issues have long concerned countries around the globe, as they do the Chinese government at all levels. In particular, preventing crop diseases and insect pests is not only crucial for increasing food production but also effective for reducing latent agricultural economic losses and facilitating accurate predictions of future grain yields. Current methods for preventing crop diseases and insect pests are still heavily reliant on manual observations by experienced farmers, and they suffer from a long-term shortage of professional agricultural technicians (He et al., 2012; Parsa et al., 2014). Faced with hundreds of millions of Chinese farming households, having only approximately 550,000 Chinese national agricultural technology extension agencies are far from sufficient (Zhang et al., 2016). Furthermore, (i) a large age gap among agricultural technicians, (ii) a lack of pest-recognition staff in each county-level plant protection station, and (iii) differing field experiences are causing a low cover density of experts specializing in pest identification and a lack of unified pest-identification criteria, thereby leading to the blind application of pesticides and serious environmental pollution (Yu, 2021).

Automatic pest identification originated from combining insect morphology with traditional machine-learning algorithms (Watson et al., 2004; Murakami et al., 2005). However, despite most researchers still placing heavy emphasis on machine-learning-based pest classification, automated pest detection based on deep learning has grown rapidly in recent years. Many researchers have used portable probes with digital cameras (Wang et al., 2021) and stationary light traps (Liu et al., 2020; Dong et al., 2021; Du et al., 2022) to automatically identify over a dozen types of tiny pests by means of artificial intelligence. Pest detection offers more semantic information with which to carry out real-world farming tasks, such as object-detection-based swarm counting (Li et al., 2022) and similar pest detection (Wang et al., 2022), whereas pest classification fails to recognize and locate multiple unknown categories of pests in a single image simultaneously. Therefore, pest detection is much more practical for precise pesticide application and pest control, and it helps agricultural plant protection experts deliver accurate treatments to control and avoid the occurrence of larger-scale pest outbreaks as early as possible.

However, current deep-learning-based methods require sufficient data to build a structural minimization model and to support cross-domain model adaption, while machine-learning-based methods demand complex hand-crafted feature descriptors and controlled laboratory backgrounds (Ngugi et al., 2021). To the best of our knowledge, little attention has been paid to those rarely collected but still harmful insect pest species whose samples are difficult to collect because of geography, season, frequency, and pest mobility (Wang, 2021). Moreover, it is difficult for even many images taken continuously from a single camera angle to fully reflect the semantic information of insects because images that are helpful for distinguishing pest species are often only a few representative images taken from multiple angles, such as of the fronts, sides, backs, and abdomens of pests (Huo and Tan, 2020). Therefore, it would be meaningful to discover a novel class with only a few instances (i.e., 10, 15, or 20 shots) (Wang et al., 2020a; Parnami and Lee, 2022). Until high-performance few-shot conceptual models that can be trained quickly become available, customization to collect big data for different scenarios is a reality that the artificial-intelligence community must face (Zhang et al., 2022). To solve this problem, we may have to start from scratch with data structure, logic causality, various invariants of vision, and compositional concept learning, among other topics, and introduce prior knowledge to auxiliary model training.

On the other hand, introducing few-shot learning technology would make it possible to detect rarely collected pest categories with just a few available samples, which would greatly reduce the cost of manual labeling through a semi-supervised automatic labeling process in which only a small amount of manual verification and calibration would have to be done by agricultural technical experts in the later stages. In addition, it would contribute to establishing a rapid response mechanism for invasive alien pests.

The contributions of this paper are summarized as follows:

1. We introduce a prior-knowledge auxiliary architecture for few-shot pest detection in the wild, which allows us to detect rarely collected pests with extremely few available samples.
2. Based on insect taxonomy, we built a new hierarchical FSIP52 data set for few-shot pest detection in natural scenarios. It could be a valuable supplementary data set for the Intellectual Plant Protection and Pest Control Community when combined with the IP102 data set (Wu et al., 2019).
3. We introduce a pest ontology relation module that is composed of a multi-relation detector and a correlation softmax loss function to incorporate prior knowledge for feature discrimination and representation. These blocks allow us to implement multi-task joint training on our model explicitly and implicitly.

## 2 Related work

### 2.1 Pest recognition

For more than a decade now, many researchers have developed various machine-learning-based pest identification methods. Larios et al. (2008) proposed a method for identifying stonefly larvae based on the scale-invariant feature transform, and it achieved a classification accuracy of 82% on four types of stonefly larvae. Wen and Guyer (2012) developed an image-based method for the automated identification and classification of orchard insects using a model that combined global and local features, and it achieved a classification performance of 86.6% on eight species of orchard field insects. Kandalkar et al. (2014) designed a pest identification procedure based on saliency map segmentation and discrete-wavelet-transform feature extraction, and utilized it in classifying pest categories using shallow back-propagation neural networks. These types of algorithms use close-up images of pest specimens in a restricted background to recognize common insects and pests, but they also require a high degree of expertise in hand-crafted feature design and parameter selection for empirical formulas. Currently, deep-learning algorithms based on large-scale data have replaced traditional pest-identification algorithms. By combining low-level and high-level contextual information of images, they have made amazing progress in identifying the pain points of detecting tiny pests and have realized the value of implementing and applying modern pest-identification algorithms. Liu et al. (2020) implemented an approach for large-scale multi-class pest detection in a stationary light trap, which could detect 16 classes with a deep-learning-based automatic multi-class crop-pest monitoring approach using hybrid global and local activated features. Wang et al. (2021) used 76,595 annotations containing ambient temperature, shooting time, and latitude and longitude information to detect *Petrobia latens*, *Mythimna separata*, and *Nilaparvata lugens* (Stål) with a smart phone in a complex field scene. However, existing deep-learning pest-recognition methods are focused mainly on identifying over a dozen of the most common pest species, for which large-scale samples of each species are required, thereby failing to meet the need for rarely collected pests. Meanwhile, pest images in most research (Li and Yang, 2020; Li and Yang, 2021) have been taken in a structured environment, such as a stationary light trap, instead of in sophisticated wild settings that are more suitable for practical applications. Therefore, being able to identify and detect novel pest classes using fewer data would make it possible to help agricultural technicians and amateur entomologists by providing them with a one-on-one expert insect encyclopedia-style service.

### 2.2 Few-shot learning

In the real world, conventional deep neural networks have always suffered from sample scarcity and the high cost of acquiring labeled data. This challenge indirectly gave rise to few-shot learning, which is generally regarded as the method of training a model to achieve good generalization performance in the target task based on very few training samples. In the fine-tuning stage, there are new classes that have never been seen before, and only a few labeled samples of each class are available; then in the testing process, when faced with new categories, the task can be completed without changing the existing model. Few-shot learning is divided into transductive learning and inductive learning, and all the models discussed herein correspond to inductive learning, in which there are three main methods, namely, meta-learning, metric learning, and transfer learning. Most few-shot classification and detection methods are based on fine-tuning (Fan et al., 2020; Kang et al., 2019; Sun et al., 2021; Wang et al., 2020b; Xiao and Marlet, 2020), and many experiments have shown that fine-tuning offers substantially improved prediction accuracy (Chen et al., 2019; Dhillon et al., 2019; Chen et al., 2020). Dhillon et al. (2019) found that a five-way one-shot fine-tuning increased accuracy by 2%–7%, while a five-way five-shot fine-tuning also increased accuracy by 1.5%–4%. Analogous conclusions have also been drawn in another work (Zhuang et al., 2020). This method is simple but useful, and its accuracy is comparable to that of other sophisticated state-of-the-art (SOTA) meta-learning methods (Li and Yang, 2020). In methods based on fine-tuning, images in the query and support set are mapped to the feature vectors, then the similarities between the query and support images in the feature space are calculated, and the final recognition result is determined by the highest similarity; thus, the model is fine-tuned efficiently even with a limited sample.

### 2.3 Few-shot pest detection

Research on identifying insect pests and crop diseases based on few-shot learning began in 2019. Li and Yang (2020) implemented metric learning in the few-shot detection of cotton pests and conducted a terminal realization with a field-programmable gate array (FPGA). Li and Yang (2021) provided the Intellectual Plant Protection and Pest Control Community with a task-driven paradigm for meta-learning in agriculture, but it only includes 10 types of close-up pests and plants in low resolution with few-shot classification configuration, which is far from real-world conditions. Yang et al. (2021) used salient-region detection and center neighbor loss to detect insects in complex real-world settings, but the approach focused on only visual features within images and did not introduce prior information to aid detection even with few samples. Yang et al. (2021) also used the iNaturalist open-source data set provided by Google, but this still includes many images whose backgrounds are not natural, generally simple backgrounds such as desktops, cement floors, and specimen trays. Moreover, their samples were collected mainly against strictly controlled laboratory backgrounds or simple natural backgrounds, and they lacked visual external features. In summary, this field is still in its infancy, aiming to identify more novel pests at low data cost.

## 3 Data preparation

Insecta is the largest class in the animal kingdom, whose number of known species exceeds 850,000, accounting for four fifths of all animals. Within Insecta, nine orders are closely related to agricultural production: Orthoptera, Thysanoptera, Homoptera, Hemiptera, Neuroptera, Lepidoptera, Coleoptera, Hymenoptera, and Diptera. In this paper, all pest species are represented by adults.

Conventionally, almost all image-classification and object-detection tasks are pretrained on the data sets provided by the ImageNet Large Scale Visual Recognition Challenge (ILSVRC) or the Microsoft Common Objects in Context (COCO) Detection Data set in order to obtain basic object features and increase the models’ generalization ability. Although these prestigious sponsors try their best, their baseline data sets still contain very few images of pests or insects. In this case, we substituted the ImageNet pre-trained data set with the IP102 and few-shot object detection (FSOD) data sets (Fan et al., 2020). IP102 (Wu et al., 2019) is an insect baseline data set that contains 18,974 images with 22,253 annotations for object detection, making it a fairly good replacement for COCO and ImageNet (Krizhevsky et al., 2017).

**Figure 1:**
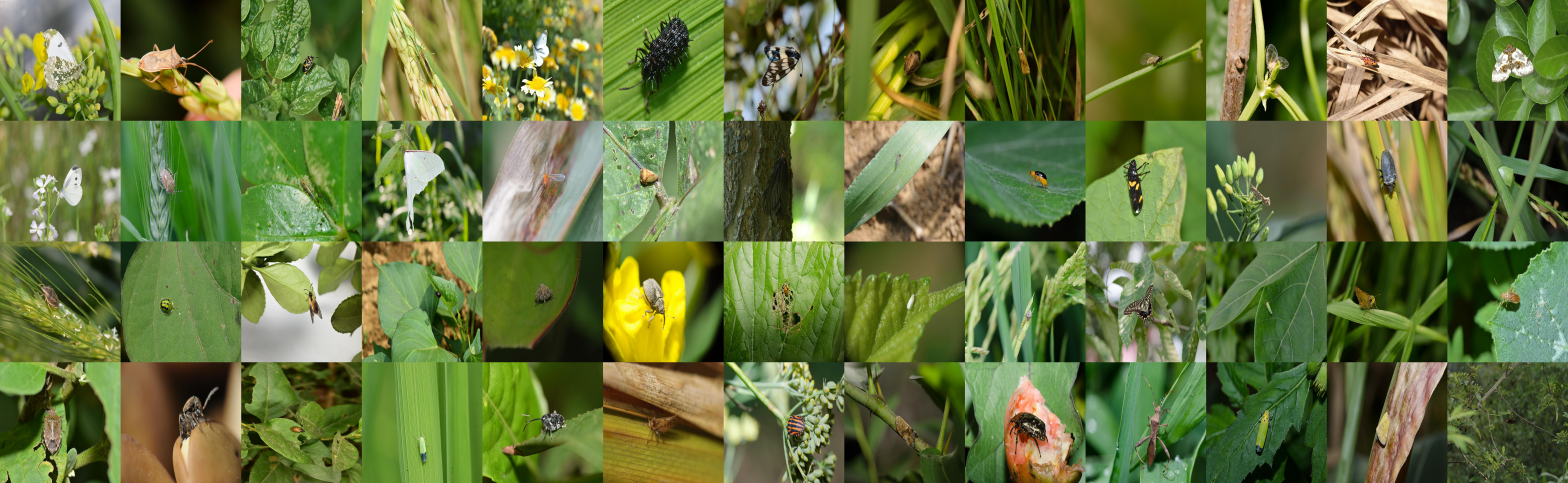
Representative demonstration images of each category in the FSIP52 data set.

However, IP102 is collected by web crawlers through common Internet image search engines such as Google, Flickr, and Bing, so it consistently suffers from poor resolution, rough annotation, improper size, and copyright watermarks. As a supplement, our FSIP52 data set contains 1,918 high-quality images that were carefully annotated and manually reviewed by pest-identification experts at the Anhui Academy of Agricultural Sciences and the Yun Fei Company, aiming to improve the signal-to-noise ratio of characteristic information in real-world pest samples with high consistency. It comprises 52 rarely collected adult agricultural and forest fruit-tree pest species with different natural backgrounds in the wild, with only dozens of samples for each pest category on average. **Figure 1** gives an intuitive visual demonstration of each category in the FSIP52 data set. The pests in each vignette are in different complex natural settings and vary in size and pose, indicating that the FSIP52 data set is very challenging. After removing the categories of IP102 that overlapped with our FSIP52, we integrated the remaining categories of IP102 as our pre-trained data set. Thus, we are able to fine-tune our model with the FSIP52 split to detect the minority pests fairly.

Few-shot object detection is quite different from general object detection methods. Few-shot learning is the process of method of training a model to achieve good generalization performance in the target task based on very few training samples. Cross-domain problems are inevitable, but they can be alleviated by constructing a source data set that is as similar to the target domain as possible. As noted by Sbai et al. (2020), base data set design is crucial for few-shot detection, and typically, it is always more important than the small improvements brought by a complex learning algorithm. Therefore, we carefully designed the base data set size and similarity to test classes and trade off between the numbers of classes and images per class. Furthermore, the degradation of plant–pest cross-domain few-shot classification performance shows the necessity of a scientifically designed data set.

**Figure 2:**
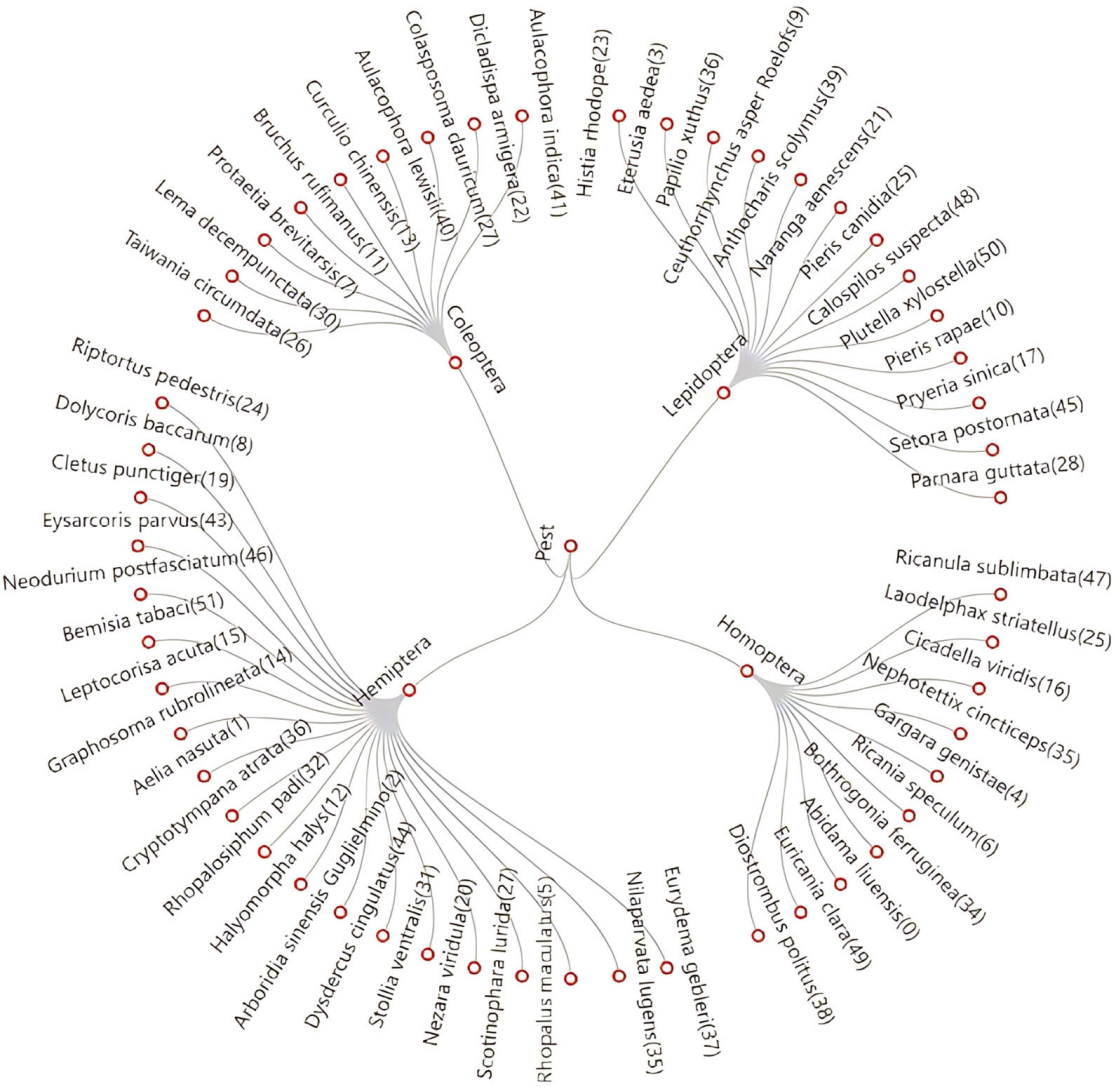
The hierarchical taxonomy of FSIP52 is explicitly stratified into four superclasses, namely, Homoptera, Hemiptera, Lepidoptera, and Coleoptera and 52 subclasses that follow the division of the pest class family. The numbers in brackets after the name of each class of insects indicate the category ID in FSIP52.

Because pest-victimized crops have complex and changing backgrounds and each pest may harm various crops, it is difficult to encode crop information as effective auxiliary information to guide the model learning. On the other hand, because insect taxonomy reveals inherent connections and provides the respective characteristics of texture and shape of various insect pests, we designed the hierarchical FSIP52 data set based on prior human knowledge and proposed a corresponding hierarchical classifier in our model. FSIP52 is divided explicitly into four super classes and further divided into 52 subclasses. The numbers in brackets after the name of each class of insects indicate the category ID in FSIP52. At the same time, we also find no intersection between our data set and 27 common stationary-light-trap agricultural pest classes that appear in Jiao et al. (2022) and belong to the rarer pest species in the data set. Nevertheless, the FSIP52 data set contains various sizes and poses, and our pre-trained data set and base class data set include three of China’s top 10 most harmful, invasive insect species in agro-ecosystems (Wan and Yang, 2016), which indicate that ours is a non-trivial practical approach to preventing the invasion of foreign insect pests. For more details, see **Figure 2**.

## 4 Proposed methodology

**Figure 3:**
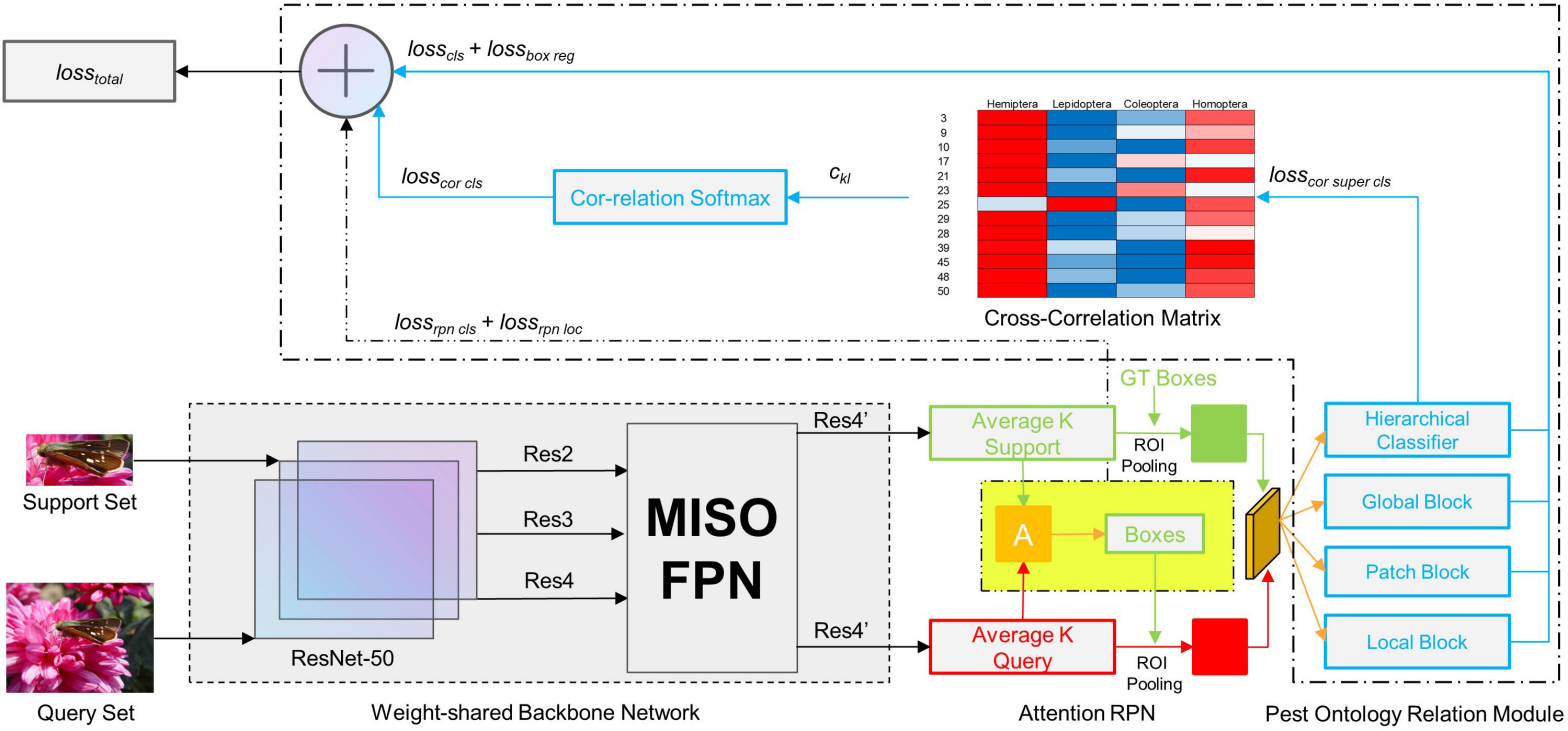
Framework of the proposed few-shot pest detection network.

The overall proposed architecture is shown in **Figure 3**. We designed our framework based on the classic Faster R-CNN framework just like other fine-tuning-based few-shot detection networks. The weight-shared backbone network extracts and shares the features of the support and query images *q_s_*, with *D*_*b*_∩*D*_*n*_=∅ . Normally, we use ResNet-50 as our backbone network and a multi-input single-output (MISO) feature pyramid network (FPN) (Lin et al., 2017; Chen et al., 2021) to introduce multiple receptive fields, aiming at the target scale imbalance problem of custom data sets. Attention region proposal network (RPN) focuses on a given support set category and filters out the target candidate frames of the other categories. Attention RPN is designed to filter out object proposals in other categories by focusing on the given support category. Support features are pooled equally into a 1×1×*C* vector, and a depth-wise cross-correlation calculation is then performed with the query features, the output of which is used as the attention features, which are fed into the RPN to generate recommendations.

For *K*-shot training, we obtain all the support features through the weight-shared network and use the average feature across all the support images belonging to the same category as its support feature. When testing, when each query image is given, these support features can be used for classification and positioning (equivalently, each test sample is a query image, which is shared by all the support images of the query image). The essence of the association between the support feature and the query feature is to use the given support image and label information to find objects with similar features in the query image and provide their approximate spatial positions. For *N*-way training, we add an *N*-1 support set branch extension network structure, where each branch has an independent attention RPN module and a multi-relation detection module.

### 4.1 Multi-relation detector

The multi-relation detector has three separate blocks: global block, local block, and patch block. Global block is used to learn the depth feature mapping information of global matching. Local block is aimed at learning the channel-by-channel spatial feature inter-correlation between the support set and candidate areas of the query set. Patch block is used to learn the similarity of the deep nonlinear metric between pixel blocks. These three subblocks calculate the similarity for each candidate area of the query set and then compare their fusion with the task threshold.

### 4.2 Hierarchical classifier and cross-correlation matrix

Fan et al. (2020) were unable to make good use of multi-source category information. Rather than using labels directly, samples were re-coded and their categories were predicted by fusing multiple feature similarities and scoring against a preset task threshold. This is essentially a clustering method by means of a specific distance measure. It would work between horses and sheep therein were similar to simple rigid bodies, and the difference between them in terms of external characteristics would still be quite obvious. However, insect pests are typically nonrigid, and insects are diverse and varied, belonging to the arthropod group of invertebrates. This paper expands the aforementioned approach by incorporating pest ontology relation module. By fusing internal and external visual information derived from the image-level pest features and hierarchical insecta information derived from prior human knowledge, multi-category information is encode to directly supervise the model optimization. Therefore, the primary difficulty in detecting pests with few samples lies in the classification of similar pest categories rather than in their localization.

**Figure 4:**
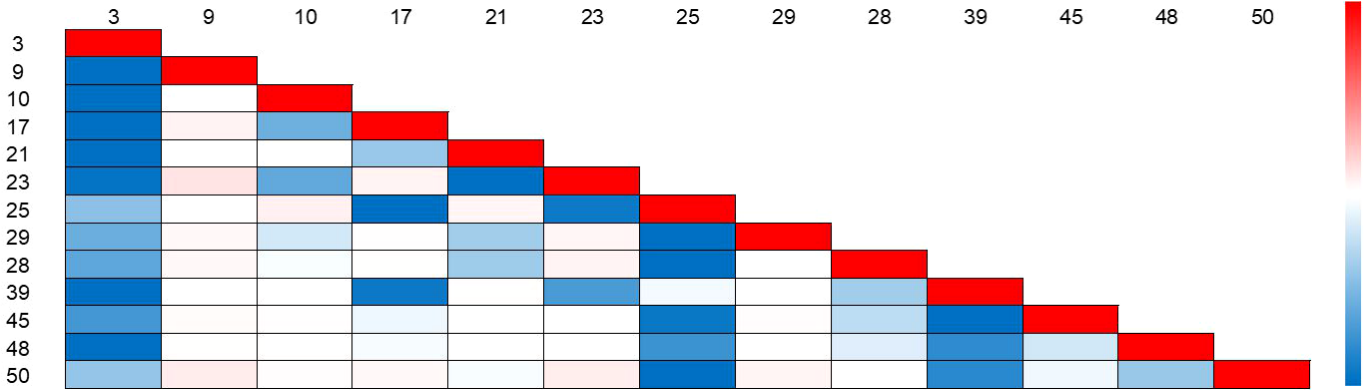
Internal dHash similarity of Lepidoptera support instances in the FSIP52 data set. The redder the heat map color block, the higher the visual similarity.

Prior knowledge derived from Insecta guides us to build a hierarchical classifier. With this, we can reduce the range of class predictions through prior human knowledge and focus more on the accuracy of classification tasks for similar classes of pests in different classes of the same order. The method of image similarity calculation has a great impact. Current few-shot detection methods (Li and Yang, 2021; Sun et al., 2021) use the Euclidean distance and the cosine similarity as the metric for the feature distance. As the dimensionality of the data increases, the maximum and minimum Euclidean distance and the cosine similarity approach zero, which makes distinguishing impossible. The Euclidean distance function and the cosine similarity function lose their meanings in a high-dimensional environment. Alternatively, we use the differential hash algorithm to encode image-level visual features, which is essentially a gradual perceptual hash algorithm combining the advantages of an average hash algorithm and a perceptual hash algorithm. We retain recognizable features at the image level through cross-correlation matrix. The internal dhash similarity of Lepidoptera support instances in the FSIP52 data set is shown in **Figure 4**. We assume that the similarity value between the same categories is 1. We find that although the similarity between different categories within the same superclass varies, their difference in similarity is not significant. Therefore, the problem of distinguishing similar pests remains a big challenge for the performance of few-shot pest detection.

The calculation phases of the cross-correlation matrix elements are as follows. First, we calculate the pairwise differential hash image similarity between each support set image of two random subclasses, *c_i_* and *c_j_*, affiliated to the same superclass, *c_l_*, to obtain the mean average dhash image similarity, *c_kl_*. In particular, when *k* and *l* are strictly affiliated in prior human knowledge, we have *p_l_* = 1; otherwise, the correlation softmax is degenerated. The purpose of this is to distinguish pests with high similarity within the same superclass by increasing the hyperplane distance between different subclasses belonging to the same superclass through loss function design. Also, the subclass distance between different superclasses is widened by having different superclasses. Thus, we fill the cross-correlation matrix with *c_kl_*.

### 4.3 Total loss function design and correlation softmax

The total loss function (*loss_total_*) deployed in the training process is defined in Equation (1).


(1) $$\begin{array}{l} loss_{total}=loss_{cls}+loss_{box\;reg}+loss_{rpn\;cls}+loss_{rpn\;loc}+loss_{cor\;cls}+loss_{cor\;super\;cls}, \end{array}$$

where *loss_boxreg_*, *loss_rpncls_*, and *loss_rpnloc_* are typical loss-function terms in Faster R-CNN; *loss_cor super cls_* is the label-smooth cross-entropy function; and *loss_cls_* is the loss sum of multi-relation detector.

*loss_cor cls_* with correlation softmax $\alpha_{k}^{*}$ is formulated as Equation (2).


(2) $$\begin{array}{l} loss_{cor\;cls}\left(s\right)=-\beta \sum_{k=1}^{m}p_{k}\log(\alpha_{k}^{*}),\\ \alpha_{k}^{*}=\frac{e^{z_{k}}}{\sum_{l=1}^{C}(2-p_{l})\left(1-c_{kl}\right)e^{z_{l}}+e^{z_{k}}}, \end{array}$$

where *β* is a scale variant that balances the numerical magnitude of the correlation softmax loss-function terms with other original loss-function terms, but it does not differentiate between easy and hard examples. Initially, we set *β = 0.25*, but *β* would be optimized after repeated experiments and changes in the data set. Through the correlation softmax function, the original softmax suppression effects between confusing pairs are weakened.

*p_k_* denotes the label of class *k* regarding bounding box *s*.

*c_kl_* is the mean average image similarity between classes *k* and *l*. Conventionally, a simple and intuitive approach would be to transform multiple binary classification problems and fuse the results, but that neglects the relationships between labels because the regular softmax loss function has exclusive semantics between labels. $\alpha_{i}^{*}$ outputs logits of correlation softmax.

Output: $P=\left({\hat{c}}_{k},{\hat{c}}_{l},{\hat{p}}_{l}\right),\;c_{k}\in \left\{0,1,2,3,\ldots ,51\right\},\;c_{l}\in \left\{0,1,2,3\right\},\;p_{l}\in \left\{0,1\right\}$

If there is a hierarchical relation between subclass *i* and its superclass *j*, then *p_l_* is set to 1, otherwise, it is set to 0.

The original Faster R-CNN loss function defined in Girshick (2015) is shown as Equation (3) and Equation (4).


(3) $$\begin{array}{l} L\left(p_{i},t_{i}\right)=\frac{1}{N_{cls}}\sum_{i}L_{cls}\left(p_{i},p_{i}^{*}\right)+\lambda \frac{1}{N_{reg}}\sum_{i}p_{i}^{*}L_{reg}\left(t_{i},t_{i}^{*}\right), \end{array}$$

Where λ = 1 and


(4) $$\begin{array}{l} L_{cls}\left(p_{i},p_{i}^{*}\right)=-\log\left(p_{i}^{*}*p_{i}+\left(1-p_{i}^{*}\right)*\left(1-p_{i}\right)\right) \end{array}$$

## 5 Evaluation metrics

### 5.1 Few-shot detection metrics

To better explain and illustrate the performance of our proposed model, we briefly describe the evaluation metrics for few-shot detection. We strictly followed the three random concepts in few-shot learning, namely, random *L*-fold cross-validation, randomly selecting *N* samples, and *K* images as support sets. The *N*-way *K*-shot definition is as follows: Randomly select *N* types of samples from the meta-data set, randomly select *K*+*m* instances from each type of sample, and then randomly select *K* instances from the *K*+*m* instances of each type of sample as the support set.

To make the obtained accuracy reasonably standardized, we use the mean average precision (*mAP*) as the metric of the proposed model. The calculation of *mAP* as defined in COCO is shown in Equation (5).


(5) $$\begin{array}{l} mAP=\frac{1}{10\times N}\sum_{k=0.5:.05}^{.95}(r_{i}-r_{i-1})\times p, \end{array}$$

where *N* denotes the total number of categories. *k* denotes the IoU threshold. *r_i_* denotes the recall value corresponding to the first interpolation of the precision interpolation segment in ascending order. *p* denotes the regression value of the observation point on the smoothed Precision-Recall (PR) curve.


(6) $$\begin{array}{l} AverageRecall=2\int_{0.5}^{1}recall\left(x\right)dx\\ =\frac{2}{n}\sum_{i=1}^{n}max\left(IoU\left(gt_{i}\right)-0.5,0\right) \end{array}$$

The definition of *AverageRecall* (AR) is first proposed by Hosang et al. (2015), and it can be calculated using Equation (6). The *AverageRecall* between 0.5 and 1 can also be computed by averaging the overlaps of each annotation *gt_i_* with the closest matched proposal, that is, integrating over the *y* axis of the plot instead of the *x* axis. *x* denotes the *IoU* overlap. *IoU*(*gt_i_*) denotes the *IoU* between the annotation *gt_i_* and the closest detection proposal. AR is twice the area enclosed by the recall-IoU curve. *n* is the number of overlaps between all GroundTruth bboxes and the nearest DetectionResult bbox in each image, that is, the COCO metric of maxDets. AR is a measure of the accuracy of the positioning of the model’s detection boxes. The mean average recall (mAR) can be obtained by averaging the AR of all categories in each novel split.

## 6 Experiments

### 6.1 Implementation details

**Figure 5:**
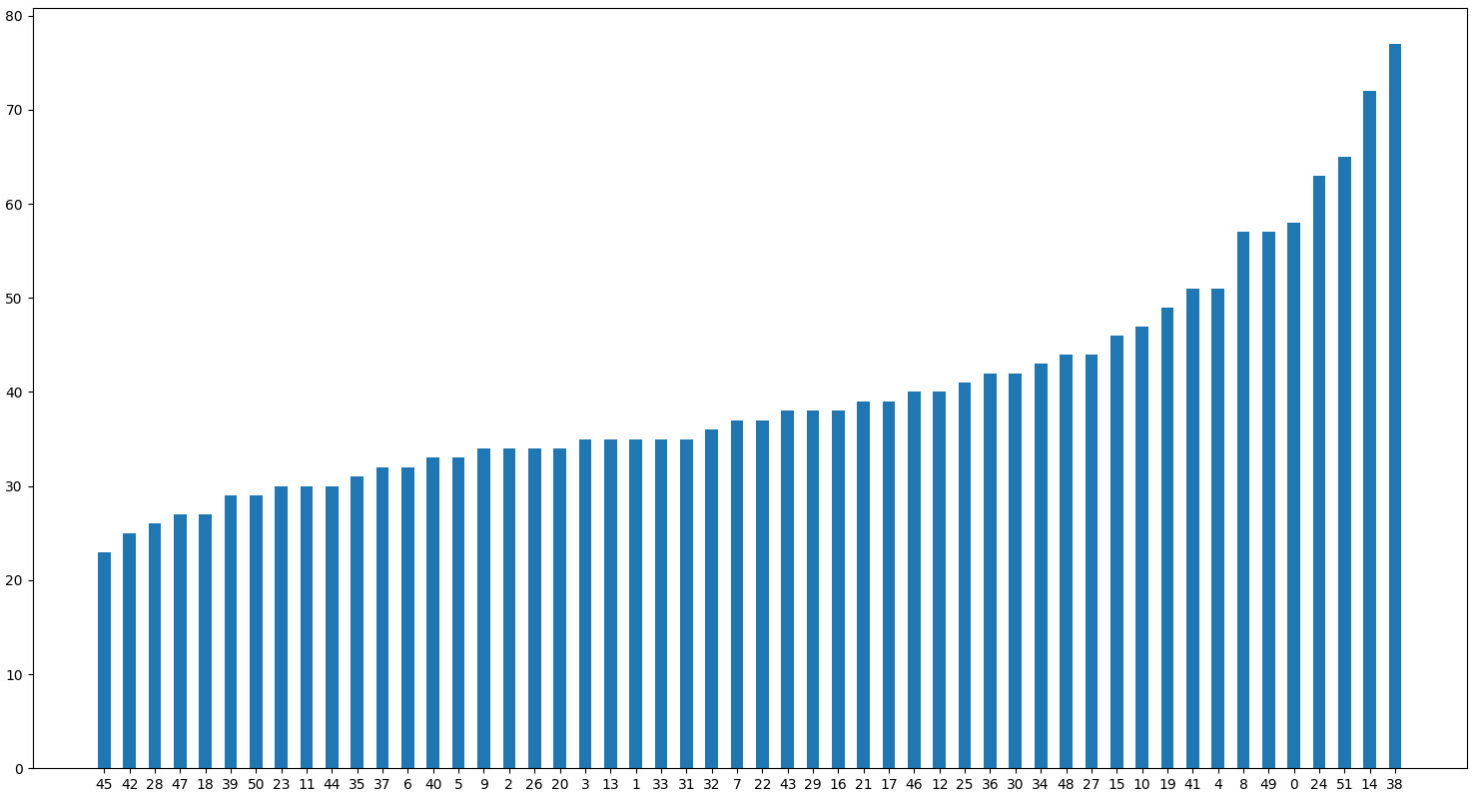
FSIP52 data set distribution is presented in ascending order according to the sample numbers. The horizontal axis represents the category ID of FSIP52 and the vertical axis represents the number of instances. Pest samples are difficult to collect due to geography, season, frequency, and pest mobility.

IP102 contains many web images and specimen images, and its image resolution ranges from 87×120 to 6034×6053 with different growth stages. There are many solid-color specimen backgrounds and single close-up images of insects in the IP102 data set, and there are many duplicate or extremely similar images of pests. We deleted some categories with very few samples, and we removed some orders of insects unrelated to what is discussed herein, specifically Hymenoptera, Diptera, Coccinellae, Acarina, Thysanoptera, Acarina, and Orthoptera. For fairness, we removed five duplicate categories between IP102 and our FSIP52 data set, namely, *Protaetia brevitarsis*, *Cicadella viridis*, *Pieris canidia*, *Papilio xuthus*, and *Nilaparvata lugens*. Finally, we removed 34 irrelevant categories from IP102, leaving IP68 to serve as our pre-trained data set. **Figure 5** and **Table 1** give more details about the distribution of the FSIP52 data set and novel class splits settings in this experiment.

**Table 1:** Detailed FSIP52 data set split experimental settings.

FSIP52	Novel split 1	Novel split 2	Novel split 3	Novel split 4
Category ID	0–12	13–25	26–38	39–51
Base class Training	1,556	1,529	1,564	1,588
Novel class fine-tuning	362	389	354	330

Since pest postures are diverse, we performed random rotation augmentations on pests in advance to compensate for the less-robust rotation invariance of a traditional convolutional neural network. The postures of pests were taken from various angles, and it is not scientific to use only similarity for supervision; the problem of pest posture can be partly solved by rotation enhancement. To analyze the proposed softmax loss and model with a hierarchical structure, we conducted extensive experiments on our well-designed FSIP52 data set. We trained our model on a computer with an Intel 9900K CPU, 128 GB of RAM, and a single NVIDIA Titan RTX GPU. In terms of software experimental conditions, we deployed our algorithm on Ubuntu 18.04.06 LTS equipped with Pycharm 2021.3 Community Edition, CUDA 11.3.1, CUDNN 8.2.1, GCC 7.5.0, Python 3.8.5, Pytorch 1.4.0, and Detectron2 0.6. For pest detection, using default anchor box settings would greatly affect the initial *IoU* value in the early training stage, resulting in the inability to screen out the optimal prediction box. Furthermore, the original IP102 data set was designed in the Visual object class (VOC) data set style, so its anchor boxes had to be re-clustered according to our data set. Moreover, the *K*-means++ clustering algorithm can randomly generate custom clustering centers, which ensures a discrete type of initial cluster center, better elevating the effect of anchor box generation. Therefore, new anchor boxes for FSIP52 were generated, including (65,86), (78,148), (119,232), (144,142), (179,339), (220,217), (292,326), (328,512), (601,698), and their re-cluster anchor aspect ratios are [0.51,0.53,0.64,0.76,0.86,0.90,1.01]. Re-clustering priori boxes helps speed up convergence.

**Figure 6:**
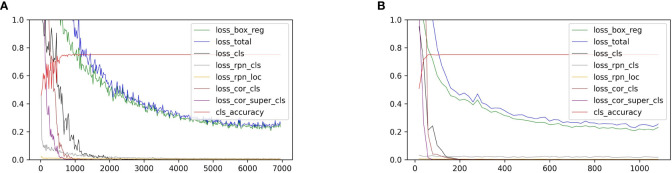
**(A)** shows the loss curves of the novel split 3 in the base-class training stage. **(B)** shows the same in the novel-class fine-tuning stage. The horizontal axis represents the number of iterations, and the vertical axis represents the loss value.

We reported our experimental results with ResNet-50 after computing the time consumption and training accuracy, although we point out that our model would perform better with other more advanced and complicated backbone networks, for example, ResNet-101 or ResNeXt. The loss curves for the base-class training stage and the novel-class fine-tuning stage are shown in **Figure 6**.

Our proposed network was trained in a class-specific and end-to-end fashion, and the original input image resolution varied from 640×480 to 3680×2456. We utilized a multi-scale training scheme to resize the input images to *x* ϵ {660×440,708×472,756×504,804×536,852×568,900×600,1000×667}. Then, the training images were resized to the same aspect ratio as the original input images, and their width and height were determined by the shorter side of the images. We trained our model for 100 epochs using the same default settings for Detectron2 in both the base-class training stage and the novel-class fine-tuning stage to ensure total complete convergence for fair comparison. An early-stopping mechanism was set to capture the best checkpoint with every 5,000 iterations, and the Dropout (Hinton et al., 2012), SoftPool (Stergiou et al., 2021), and DropBlock (Ghiasi et al., 2018) techniques were also introduced in the pre-training, base-class training, and fine-tuning stages.

In the base-class training stage, the learning rate was set to 0.001 with 100 epochs and a batch size of eight. The fraction between positive and negative samples was 0.5 and was kept the same in both the training and testing sets in both stages. Weight-shared ResNet-50 was pretrained on the FSOD data set to extract features from the support and query images, and its output features were the set {*res*2,*res*3*,res*4*,res*5}. Deformable convolution was applied in the feature-extraction and FPN stages, and the non-maximum suppression threshold in RPN was set to 0.7. The smooth L1 beta was 1/9, the *IoU* threshold in Region of Interest (ROI) head was set to 0.3, the weight decay applied to the parameters of the normalization layers was 1×10^-5^, the momentum was set to 0.937, the warm-up iterations were set to 2 epochs, the default support ways for contrastive learning branch were 2, and the ResNet-50 backbone network was frozen at *res*3. We decoupled the fully connected layers concerning both the cross-correlation matrix and the hierarchical matrix with the original Faster-RNN classifier layer. We applied Kaiming normal weight initialization (He et al., 2015) to all convolutional and fully connected layers and inputted the concatenation of the support and query features. MISO FPN outputted the *res*4 feature for further processing, and group normalization was enabled in FPN. FPN and RPN were jointly optimized in both stages.

In the novel-class fine-tuning stage, the learning rate was set to 0.001 with 100 epochs and a batch size of 12. Most pretrained model parameters or layers were frozen, while only the last few layers’ parameters were updated during the novel-class training.

### 6.2 Comparison experiments and discussion

Research on few-shot object detection has emerged in the past 2 years, and we decided to compare our method with several typical few-shot object detection networks, namely, those by Fan et al. (2020); Sun et al. (2021); Wu et al. (2020) and Wang et al. (2020b). All comparison experiments were conducted on the MMFewShot framework produced by Open MMLab and the Detectron2 framework produced by Facebook, using exactly the same experimental settings. Our model outperformed most state-of-the-art (SOTA) methods without much extra calculation.

Before fully analyzing and discussing the results of the experiments, it must be pointed out again that our custom data sets were all taken from real natural scenarios that have been strictly selected by the Yun Fei Company, Anhui Academy of Agricultural Sciences, and the Hefei Plant Protection Station pest experts, making the samples rather representative and complex. Note that insects are nonrigid bodies, and their tentacles can easily expand the bounding box unnecessarily and cause a reduction in the signal-to-noise ratio, which then leads to quite large bounding boxes. On the other hand, due to the migratory nature of some pests, close-up photography is not possible, so certain tiny pests add difficulty to the current few-shot pest detection in the wild.

In **Table 2**, our model achieves the best results on the FSIP52 data set based on a few-shot protocols of 13-way 10 shots on novel splits 1, 3, and 4 and is ahead of SOTA methods by 4%, 2.8%, and 2.2% on mAP, respectively. In **Table 3**, it is ahead of SOTA methods by 5.9%, 2.8%, and 0.6% on AP50, respectively. In **Table 4**, our model outperforms SOTA on novel splits 1, 2, and 3 by 7%, 10.9%, and 7.8% on mAR, respectively. The reason for this is that our model was especially designed for pest in wild settings. We availed of multi-task learning to design a logically interpretable prior knowledge learning task, and import the knowledge gained by human experts in the process of pest identification as supervision information to guide the network to achieve better detection performance in the case of extremely limited novel class samples. The use of cosine classifier and contrastive loss coverages very slowly in the set number of iterations by Sun et al. (2021) may not suitable for pest detection, and its coefficients are too many to be fine-tuned.

**Table 2:** FSIP52 novel classes’ mean average precision (mAP) in 13-way 10-shot settings.

Reference	Novel split 1	Novel split 2	Novel split 3	Novel split 4
Wu et al. (2020)	6.7	**22.3**	9.9	10.4
Fan et al. (2020)	12.5	15.4	16.1	11.2
Wang et al. (2020b)	11.6	12.0	12.5	11.7
Sun et al. (2021)	7.2	9.7	9.8	5.3
Ours	**16.5**	17.0	**18.9**	**13.9**

Bold values are to highlight which models achieved the highest accuracy in the different data set splits, in order to provide strong evidence of the advantages of a particular method.

**Table 3:** FSIP52 novel classes’ AP50 in 13-way 10-shot settings.

Reference	Novel split 1	Novel split 2	Novel split 3	Novel split 4
Wu et al. (2020)	17.4	**44.3**	23.9	19.7
Fan et al. (2020)	20.3	25.0	27.4	20.0
Wang et al. (2020b)	24.6	29.4	30.0	22.7
Sun et al. (2021)	16.8	21.7	22.2	13.3
Ours	**30.5**	32.9	**35.9**	**23.3**

Bold values are to highlight which models achieved the highest accuracy in the different data set splits, in order to provide strong evidence of the advantages of a particular method.

**Table 4:** FSIP52 novel classes’ mean average recall (mAR) in 13-way 10-shot settings.

Reference	Novel split 1	Novel split 2	Novel split 3	Novel split 4
Wu et al. (2020)	38.5	52.4	36.4	39.1
Fan et al. (2020)	57.6	55.0	56.8	**57.3**
Wang et al. (2020b)	42.2	42.4	40.8	40.5
Sun et al. (2021)	37.3	37.9	40.5	37.3
Ours	**64.6**	**65.9**	**64.6**	55.9

Bold values are to highlight which models achieved the highest accuracy in the different data set splits, in order to provide strong evidence of the advantages of a particular method.

Nonetheless, note that our model trails that of Wu et al. (2020) by 5.3% and 11.4% on mAP on Novel split 2. A comparison of each category in Novel split 2 shows that the model of Wu et al. (2020) leads our model in categories 13, 14, 18, 19, 21, and 22 by 20.6%, 6.7%, 1.5%, 10.3%, 13.6%, and 29.7%, respectively. Yet the mAR of our model prevails over that of Wu et Al. by 13.5%. We attribute this to the presence of extra-large and tiny targets in these categories; the predominance of frontal and abdominal photographs of the pests, which does not capture the most recognisable parts of the pests; and the fact that our model does not have a re-weighted strategy for these multi-scale positive samples through especially designed reinforcement block. Although we slightly underperformed compared to Fan et al. by 1.4% in the Novel split 4 mAR comparison, we achieved comparable performance to SOTA in preventing missed detections and were 2.7% and 3.3% ahead of that of Fan et al. in mAP and AP50, respectively, which are often more important in practice than mAP and AP50. Finally, despite the fact that our performance improved compared with the SOTA methods mentioned, we still have a long way to go to be qualified for real-world agricultural production missions.

## 7 Conclusion

In this paper, a few-shot insect pest detection network is introduced to detect rarely collected pest species. Its novelty lies in combining the hierarchical semantic relationship between superclasses and subclasses according to insect taxonomy, guiding our model to better learn novel concepts through causal intervention, especially when the novel class samples are extremely limited. A new hierarchical data set FSIP52 for few-shot pest detection in natural settings is built based on insect taxonomy. It is emphasized that the presented few-shot pest detection network achieves comparable performance to several representative few-shot detection algorithms in FSIP52 data set through incorporating pest ontology relation module designed specifically for hierarchical structure matching in the proposed framework, and we point out that it could be extended to other similar practical scenarios with hierarchical structures. Last but not the least, apart from the developed fine-tuning-based object detection algorithms, there are other branches of few-shot learning methods (e.g., cross-domain and meta-learning) that are still at a relatively preliminary stage and are quite worthy of follow-up research. The present work highlights a new entry in the field of few-shot pest detection.

## Data availability statement

The data sets presented in this article are not readily available because permission must be obtained from the head of the laboratory and the supervisor. Requests to access the data sets should be directed to mydream@mail.ustc.edu.cn.

## Author contributions

XW is responsible for the methodology, model building, data set construction, experimental implementation and the first draft of this paper. JD is responsible for the conceptualized guidance of paper writing and the discussion of the overall architecture. CX programmatically pointed out the initial research direction. SW took part in code debugging and idea discussion. XM participated in discussions on initial concept formation and model validation. KL is responsible for the verification of model formulas and manuscript revision. SD proofread the whole paper. TC provided basic agricultural knowledge consultation. All authors contributed to the article and approved the submitted version.
